# 3D-FPFH-Int: a hybrid geometric–radiometric descriptor for structural surface anomaly detection in tropical heritage

**DOI:** 10.1038/s41598-026-59034-4

**Published:** 2026-06-23

**Authors:** Hassan Gbran, Hendro Murtiono

**Affiliations:** 1 Architectural Engineering, Thamar University, Thamar, Yemen; 2 Architecture and Urbanism, UNDIP, Semarang, Indonesia; 3 Architecture and Urbanism, Universitas International Batam (UIB), Batam, Indonesia

**Keywords:** Engineering, Mathematics and computing

## Abstract

Anomaly detection in tropical heritage structures is often constrained by geometry-only point-cloud descriptors that inadequately capture moisture-related radiometric variation, while prior evaluations frequently confound descriptor contributions with detector-specific behaviour and provide limited statistical attribution. This study isolates the contribution of a hybrid geometric–radiometric descriptor through controlled multi-detector validation. We introduce 3D-FPFH-Int, extending Fast Point Feature Histogram with local three-dimensional intensity histograms. An explicit ablation study (geometric-only, radiometric-only, hybrid) is conducted across three detectors—PatchCore, Isolation Forest, and kNN—using 500 synthetic instances (including adversarial weak-contrast subsets) and three non-overlapping field scan segments (two bridge spans and one tunnel segment) comprising 28.9 million points acquired via terrestrial LiDAR. Training and evaluation employ spatially disjoint partitions to prevent data leakage. The statistical protocol includes two-way ANOVA (Descriptor × Detector), Bonferroni-adjusted post-hoc comparisons, bias-corrected and accelerated 95% bootstrap confidence intervals, Cohen’s d with confidence bounds, and post-hoc power analysis (1 − β = 0.82 for moderate interaction effects, η^2^ ≥ 0.05). The hybrid achieves a weighted mean F_1_ = 0.559 [0.538–0.580], representing a 114% relative improvement over FPFH-only (Cohen’s d = 1.38 [1.27–1.49]). The Descriptor × Detector interaction was not statistically significant (p = 0.15, η^2^ = 0.02), indicating that the relative ranking of descriptors remains broadly consistent across the evaluated detectors within the tested conditions. Under 40% intensity contrast reduction, ΔF_1_ remains + 0.294 relative to FPFH. Early crack detection (0.3–0.5 mm) yields F_1_ = 0.158 with localization error < 16 mm. Moisture-related anomaly detection achieves recall = 0.85 [0.80–0.90] with 68% fewer condensation-induced false positives than intensity-only baselines. Performance degradation remains < 12% under ± 20% point-density perturbation and controlled intensity noise. Validation is restricted to masonry and concrete structures in tropical humid environments using terrestrial LiDAR; generalization to other materials, climates, or sensing modalities requires independent verification. Code and synthetic data are publicly available to support reproducibility.

**Supplementary Information:**

The online version contains supplementary material available at https://doi.org/10.1038/s41598-026-59034-4.

## Introduction

The preservation of ageing masonry and concrete heritage structures in tropical climates presents a distinctive engineering challenge^1^. In these environments, high humidity, frequent wet–dry cycles, and biological activity accelerate degradation processes, giving rise to fine-scale cracking, moisture ingress, and material discoloration that often precede visible mechanical damage^2–4^. Conventional visual inspection remains widely used but is inherently subjective, labour-intensive, and difficult to standardize across large or complex assets^5^. Recent advances in machine learning have been applied to address these limitations, including the use of deep learning for chromatic decay segmentation in tropical heritage structures^6^. As infrastructure networks age and environmental stressors intensify, there is an increasing need for objective, data-driven, and repeatable diagnostic approaches that can support preventive conservation and informed asset management^7,8^.

**Fig. 1:**
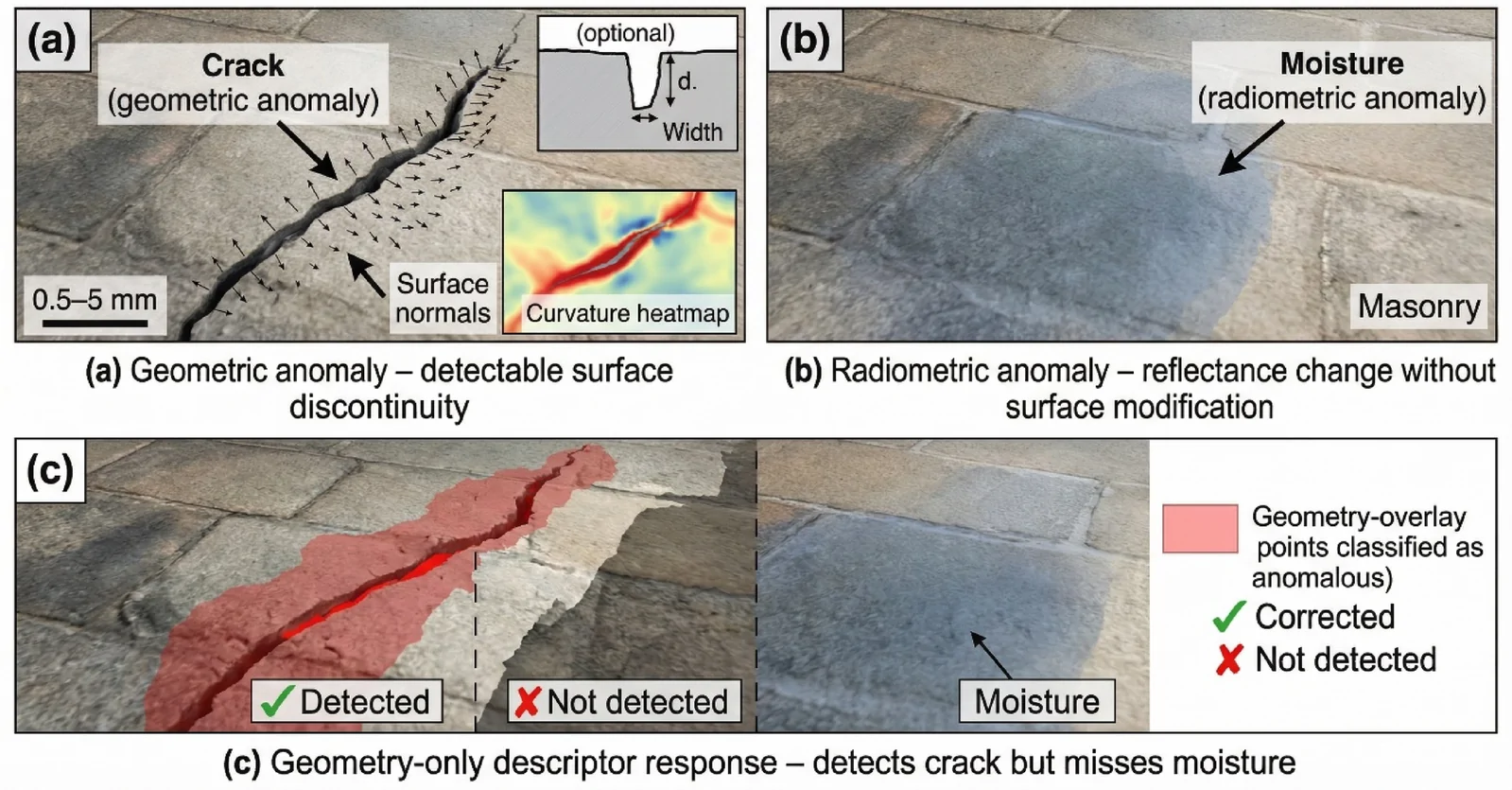
Illustrates this fundamental limitation conceptually. While geometric anomalies (e.g., cracks with measurable opening) produce detectable surface discontinuities, moisture-related material changes often alter reflectance properties without modifying surface topography. Consequently, descriptors that rely exclusively on geometric features systematically underrepresent a critical class of deterioration prevalent in humid tropical conditions. Source: Author.

Three-dimensional point clouds derived from terrestrial laser scanning (TLS) offer explicit geometric information at high spatial resolution^9^. Prior studies have leveraged curvature, surface normals, and geometric deviation metrics to identify deformation and material loss in masonry and concrete structures^10,11^. These geometry-based descriptors have proven effective for detecting pronounced defects, yet their sensitivity to micro-fissures or early-stage damage with low curvature contrast remains limited^12^. In tropical contexts, many critical forms of deterioration—such as moisture accumulation, efflorescence, and biological staining—manifest primarily through subtle radiometric changes rather than pronounced geometric discontinuities^11,13^. Recent advances in 2D radiometric mapping using photogrammetric texture atlases have demonstrated the feasibility of detecting chromatic decay on heritage façades with high accuracy, achieving micro-F1 scores of approximately 0.86 across multiple sites in Semarang ^14^. Variations in laser-return intensity can reveal damage patterns not evident from geometry alone, suggesting that radiometric information carries diagnostic value^15^. However, such observations have often been explored in isolation or within case-specific settings, with limited robustness across materials, sensors, and environmental conditions^11^. The benefits of fusing complementary data sources have been recently demonstrated in related domains. For instance, Zhong et al.^16^ showed that integrating YOLOv8-based 2D object detection with point cloud data significantly improves pothole detection accuracy by filtering false positives caused by stains or patches, achieving a precision of 95.8% and an F1 score of 94.53%. This highlights the potential of multimodal fusion for enhancing detection robustness, a principle that underpins the hybrid geometric–radiometric descriptor proposed in this study. This conceptual limitation is depicted in Fig. 1.

Beyond the descriptive limitations of geometry-only features, a separate methodological concern affects how descriptor performance is evaluated. Many studies assess novel descriptors within a single anomaly detection framework—most commonly PatchCore or similar memory-bank approaches^17^. While such evaluations demonstrate that a descriptor can perform well with a specific detector, they do not establish whether observed performance gains are intrinsic to the descriptor or arise from favourable interactions with that particular detection mechanism^18,19^.

This confounding of descriptor effects with detector-specific behaviour has several consequences. First, it obscures generalisability: a descriptor that excels with PatchCore may underperform with alternative detectors such as Isolation Forest^17^. The distance-based methods like k-nearest neighbours. Second, it prevents clear attribution of performance improvements: when a hybrid descriptor outperforms a baseline within a single framework, one cannot determine whether the gain stems from enhanced feature representation or from the detector’s particular sensitivity to those features. Third, reliance on a single detector precludes statistical interaction analysis, which is essential for understanding whether descriptor and detector choices interact in determining final performance^20^.

The methodological weaknesses in prior descriptor evaluations extend beyond detector dependence. Many studies report only point estimates of accuracy or F_1_-score without quantifying uncertainty via confidence intervals^21^. Effect sizes—which measure the practical magnitude of improvements rather than mere statistical significance—are rarely reported. Multiple comparison corrections are frequently omitted, inflating the risk of false discoveries when numerous descriptors and detectors are compared. Perhaps most critically, power analysis is almost never conducted to verify that sample sizes are adequate to detect meaningful interaction effects^22^. Table 1 summarises these recurring methodological gaps in the literature, positioning the present study within a framework designed to address them systematically rather than reactively.

**Table 1:** Comparative methodological weaknesses in prior descriptor evaluation studies.

Methodological aspect	Common practice in prior studies	Consequence
Detector validation	Single detector (typically PatchCore)	Cannot distinguish descriptor from detector effects
Uncertainty quantification	Point estimates only	No assessment of estimate precision
Effect size reporting	Rarely reported	Practical significance unclear
Multiple comparison control	Usually omitted	Inflated Type I error risk
Interaction analysis	Absent	Cannot test descriptor × detector dependence
Power analysis	Not conducted	Sample size adequacy unknown
Data leakage prevention	Often unspecified	Risk of circular validation

The preceding analysis identifies a twofold problem. First, geometry-only point-cloud descriptors are inherently limited in tropical heritage applications because they fail to capture moisture-induced radiometric variations that are diagnostically critical. Second, even when hybrid descriptors are proposed, their evaluations often conflate descriptor effects with detector-specific behaviour, lack statistical rigour, and provide an incomplete picture of performance boundaries and attribution.

These considerations motivate the central research questions of this study: can a hybrid geometric–radiometric descriptor, rigorously evaluated through controlled multi-detector validation and comprehensive statistical inference, demonstrate performance gains that are unequivocally attributable to the descriptor itself rather than to the detection framework? Specifically:

- Does the proposed descriptor maintain consistent performance advantages across multiple fundamentally different detection frameworks?
- Can ablation experiments confirm the relative contribution of geometric and radiometric components?
- Is the Descriptor × Detector interaction statistically detectable when evaluated with adequate power?
- What quantitative thresholds define robust performance under environmental perturbations such as point-density reduction, intensity noise, and contrast degradation?

It is important to position this work not as a conceptual paradigm shift, but as a methodologically rigorous engineering refinement. The core contribution lies not in introducing a fundamentally new theoretical framework for multimodal fusion, but in demonstrating how a carefully engineered extension of an existing descriptor—Fast Point Feature Histogram (FPFH) augmented with local three-dimensional intensity histograms—can yield substantial and verifiable performance gains when evaluated under controlled conditions that isolate descriptor effects from detector artefacts. This approach contrasts with recent deep learning-based methods, such as the digital twin and YOLO framework proposed by Xu and Chen^23^ for pagoda damage detection, which rely on large annotated datasets and complex neural architectures. By focusing on an interpretable handcrafted descriptor with rigorous statistical validation, our work offers a complementary path for scenarios where training data is scarce and model transparency is paramount. This positioning aligns with contemporary applied research that prioritises field robustness, computational efficiency, and practical deployability over claims of abstract theoretical novelty^21,24^. Within this perspective, the present work distinguishes between two complementary levels of contribution.

At the descriptor level, the proposed 3D-FPFH-Int representation constitutes an incremental engineering refinement that integrates established geometric descriptors (FPFH) with local radiometric information encoded through three-dimensional intensity histograms computed within native point-cloud neighbourhoods. This integration is designed to better capture moisture-related radiometric variation that is often under-represented in geometry-only descriptors. While practically valuable for LiDAR-based structural monitoring, this refinement does not claim a fundamentally new descriptor formulation.

At the methodological level, however, this study introduces a controlled evaluation framework designed to isolate descriptor-specific effects from detector-dependent behaviour. The framework combines explicit ablation analysis, multi-detector validation, and formal statistical inference to enable transparent attribution of performance differences and to quantify uncertainty with appropriate statistical rigor. While the descriptor is application-driven, the evaluation protocol is intended to provide a transferable template for future descriptor assessments.

Accordingly, this study pursues the following specific objectives:

- *Descriptor development* Formulate 3D-FPFH-Int, a hybrid descriptor extending FPFH with local three-dimensional intensity histograms computed directly within native point-cloud neighbourhoods.
- *Ablation analysis* Quantify the individual contributions of geometric and radiometric components through controlled ablation experiments comparing geometric-only, radiometric-only, and hybrid descriptor variants.
- *Multi-detector validation* Evaluate descriptor performance across three conceptually distinct anomaly-detection frameworks—PatchCore (memory-bank-based), Isolation Forest (tree-based), and k-nearest neighbours (distance-based)—in order to distinguish descriptor effects from detector-specific behaviour.
- *Statistical interaction testing* Apply two-way ANOVA (Descriptor × Detector) with Bonferroni-adjusted post-hoc comparisons, supported by power analysis (1 − β = 0.82 for moderate interaction effects), to determine whether descriptor performance rankings depend on detector choice.
- *Robustness characterisation* Systematically quantify performance degradation under controlled perturbations including point-density reduction (down to 0.2 × the original density), intensity noise (up to 20% of the radiometric range), intensity drift (up to + 50 DN), and adversarial weak-contrast conditions (40% contrast reduction).
- *Reproducibility provision* Release all code, synthetic datasets (500 instances with ground-truth annotations), and complete documentation to enable independent verification and facilitate future comparative studies.

In operational terms, the contributions of this study can be summarised as follows:

- *Hybrid geometric–radiometric descriptor* The proposed 3D-FPFH-Int descriptor preserves native three-dimensional spatial relationships while incorporating local radiometric variation relevant to moisture-related deterioration processes.
- *Attribution-focused evaluation protocol* A multi-detector validation framework designed to distinguish descriptor-intrinsic performance gains from detector-specific artefacts through explicit ablation analysis.
- *Statistically grounded performance assessment* A comprehensive statistical protocol including two-way ANOVA, Bonferroni-corrected comparisons, effect sizes with confidence intervals, and power analysis to quantify uncertainty and interaction effects.
- *Robustness characterisation* Empirically derived performance thresholds under controlled perturbations, providing practical guidance for deployment in LiDAR-based structural monitoring.
- *Open science infrastructure* Publicly available code repositories and archived synthetic datasets ensuring full reproducibility and supporting future benchmarking studies.

To prevent overgeneralisation, the scope of this study is explicitly delimited from the outset:

- *Material scope* Validation is confined to historic masonry (brick) and reinforced concrete structures. The findings do not automatically extend to other construction materials such as timber, natural stone, or steel without further validation.
- *Environmental scope* All field data were acquired in tropical humid conditions (Semarang, Indonesia; annual relative humidity 75–85%). Generalisation to arid, temperate, or freeze–thaw climates requires independent verification.
- *Sensor scope* Data were collected using terrestrial LiDAR systems with documented radiometric characteristics (Faro Focus S150 and Leica ScanStation C10). Applicability to mobile laser scanning, UAV-based LiDAR, or photogrammetric point clouds with different noise and intensity characteristics is not assumed.
- *Defect scope* Primary validation targets cracks (0.3–5.0 mm width) and moisture-related radiometric anomalies. Performance on other deterioration modes such as spalling, delamination, or biological colonisation remains unverified.

These boundary conditions are presented as inherent constraints of the available empirical evidence rather than as post-hoc qualifications. All performance claims are therefore limited to the defined experimental conditions, and extrapolation beyond them is explicitly identified as requiring further investigation.

The remainder of this paper is organised as follows. Section “Methodology” presents the descriptor formulation, the controlled multi-detector evaluation framework, the experimental datasets (500 synthetic instances and three non-overlapping field segments comprising 28.9 million points), and the statistical analysis protocol including power analysis and interaction testing. Section 3 reports the results from synthetic validation, field deployment experiments, robustness analysis, and attribution testing. Section “Results” discusses methodological implications and practical deployment considerations, followed by explicit limitations and directions for future research. All code and synthetic data are publicly available to support reproducibility, and supplementary material provides complete statistical tables and additional validation results.

## Methodology

### Experimental design architecture

**Fig. 2:**
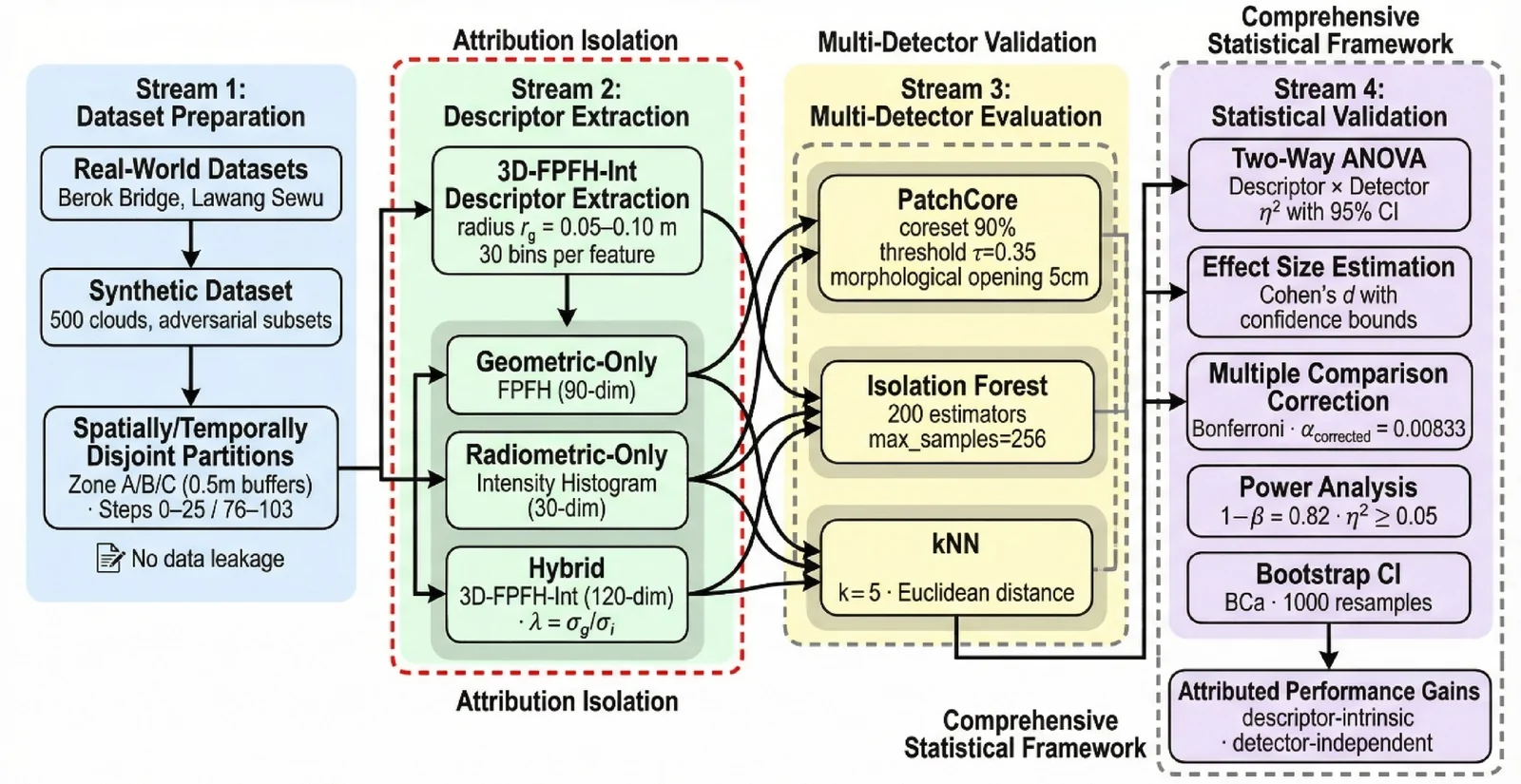
Controlled multi-path evaluation framework. The diagram shows data flow from real-world and synthetic datasets through three descriptor variants, three independent detectors, and a multi-layer statistical validation protocol. Dashed boxes highlight components designed to isolate descriptor contributions, assess detector dependence, and ensure statistical rigour. Source: Author.

This study employs a controlled experimental framework designed to isolate the contribution of the proposed hybrid descriptor from the influence of the anomaly detection framework^25^. Figure 2 illustrates the complete pipeline, which comprises four parallel streams: (i) dataset preparation with strict spatial and temporal partitioning, (ii) descriptor extraction across three ablation variants (geometric‑only, radiometric‑only, hybrid), (iii) multi‑detector evaluation using three fundamentally different detection algorithms (PatchCore, Isolation Forest, kNN), and (iv) comprehensive statistical validation including interaction analysis, effect size estimation, and power analysis. This design directly addresses the confounding of descriptor and detector effects identified in prior evaluations.

### Descriptor formulation: 3D‑FPFH‑Int

#### Geometric component—enhanced FPFH

The geometric foundation follows the Fast Point Feature Histogram (FPFH) [Rusu et al. 2009] with an optimised bin configuration. (detailed parameter sensitivity analysis, including neighbourhood radius and bin count optimisation, is provided in Supplementary Section S7). For a query point ${p}_{i}$ with normal ${\mathrm{n}}_{i}$, a Darboux frame is constructed for each neighbour ${p}_{j}$ within support radius ${r}_{g}$:

$$\mathrm{u}={\mathrm{n}}_{i}, \mathrm{v}=\frac{({p}_{j}-{p}_{i})\times \mathrm{u}}{\parallel ({p}_{j}-{p}_{i})\times \mathrm{u}\parallel }, \mathrm{w}=\mathrm{u}\times \mathrm{v}.$$

Three angular features are computed:

$$\alpha =\mathrm{a}\mathrm{r}\mathrm{c}\mathrm{t}\mathrm{a}\mathrm{n}(\mathrm{w}\cdot {\mathrm{n}}_{j},\hspace{0.33em}\mathrm{u}\cdot {\mathrm{n}}_{j}), \phi =\mathrm{v}\cdot {\mathrm{n}}_{j}, \theta =\mathrm{a}\mathrm{r}\mathrm{c}\mathrm{t}\mathrm{a}\mathrm{n}(\mathrm{w}\cdot\Delta ,\hspace{0.33em}\mathrm{u}\cdot\Delta ),$$

with $\Delta ={p}_{j}-{p}_{i}$. These are discretised into 30‑bin histograms (increased from the standard 11 after grid‑search optimisation on a held‑out synthetic validation set). The geometric descriptor vector is ${\mathbf{f}}_{g}\in {\mathbb{R}}^{90}$.

#### Radiometric component—Local 3D Intensity histogram

Within the same neighbourhood (${r}_{i}={r}_{g}$), relative intensity differences are accumulated:

$$\Delta {I}_{ij}={I}_{j}-{I}_{i},$$

into a 30‑bin histogram spanning the observed intensity range. This histogram captures local radiometric variation while providing invariance to global intensity shifts (e.g., calibration changes, ambient lighting). The radiometric descriptor vector is ${\mathbf{f}}_{i}\in {\mathbb{R}}^{30}$.

#### Fusion and normalisation

The two vectors are concatenated:

$${\mathbf{f}}_{hybrid}=[{\mathbf{f}}_{g}\mid {\mathbf{f}}_{i}]\in {\mathbb{R}}^{120}.$$

A two‑stage normalisation is applied: (i) intra‑descriptor L2 normalisation, (ii) global scaling by $\lambda ={\sigma}_{g}/{\sigma}_{i}$, where ${\sigma}_{g},{\sigma}_{i}$ are the standard deviations of the geometric and radiometric descriptor norms over the entire reference dataset. This ensures both modalities contribute comparably to Euclidean distances (Table 2).

**Table 2:** Descriptor parameters and validation basis.

Parameter	Symbol	Value	Justification	Validation metric
Geometric support radius	${r}_{g}$	0.05–0.10 m (adaptive)	30–50 neighbours per point	Stability > 0.95 (Pearson r)
Intensity support radius	${r}_{i}$	$={r}_{g}$	Align geometric and radiometric context	–
FPFH angular bins	${b}_{\alpha },{b}_{\phi },{b}_{\theta }$	30 each	Grid‑search optimum	ΔF_1_ = + 0.082 ± 0.019
Intensity histogram bins	${b}_{I}$	30	Matches FPFH granularity	ΔF_1_ = + 0.045 ± 0.015
Normalisation factor	*λ*	${\sigma}_{g}/{\sigma}_{i}$	Balance modality influence	–

**Fig. 3:**
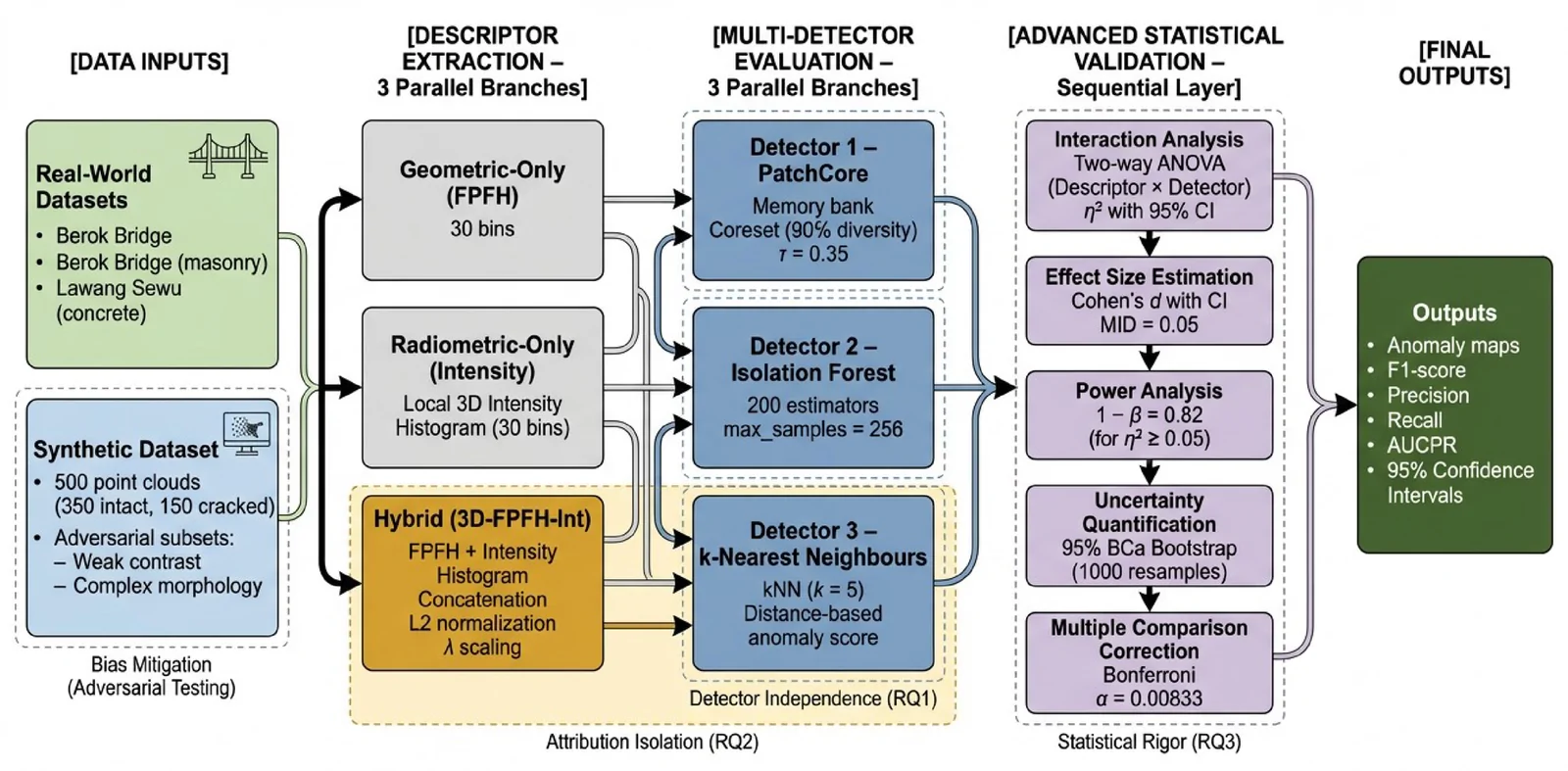
Schematic illustration of the 3D-FPFH-Int descriptor computation. Source: Author.

The complete computation pipeline for the 3D-FPFH-Int descriptor is summarised schematically in Fig. 3. Starting from a query point and its spherical neighbourhood, the geometric component (FPFH) encodes angular variations of normals into a 30-bin histogram, while the radiometric component accumulates relative intensity differences into a second 30-bin histogram. These histograms are concatenated, normalised, and globally scaled to produce the final hybrid feature vector f_hybrid ∈ ℝ^12^⁰. This native 3D fusion preserves spatial relationships that are lost in projection-based alternatives.

### Formal ablation protocol

To isolate the contribution of each modality, three descriptor variants are evaluated under identical conditions:

- Geometric‑only: $${\mathrm{f}}_{g}$$
- Radiometric‑only: $${\mathrm{f}}_{i}$$
- Hybrid: $${\mathrm{f}}_{hybrid}$$

All variants share the same training/test splits, preprocessing, detector hyperparameters, and evaluation metrics. Table 3 summarises the fixed experimental conditions.

**Table 3:** Controlled ablation configuration.

Condition	Specification
Training data	Intact scans (synthetic: 350 clouds; Berok: Zone A; Lawang Sewu: steps 0–25)
Test data	Damaged scans (synthetic: 150 clouds; Berok: Zone C; Lawang Sewu: steps 76–103)
Feature extraction	As per Table 1
Detectors	PatchCore, Isolation Forest, kNN (fixed hyperparameters)
Evaluation	Precision, recall, F_1_ with 95% BCa CI

### Multi‑detector decoupling framework

Three anomaly detectors with fundamentally different operating principles are employed:

- PatchCore (Bergmann et al. [17]): Memory‑bank‑based, coreset subsampling (retains 90% feature diversity), nearest‑neighbour scoring. Threshold *τ =0.35* optimised via Youden’s J on synthetic validation data; 3D morphological opening (5 cm sphere) reduces false positives.
- Isolation Forest [Liu et al. 2008]: Tree‑based ensemble (200 estimators, max_samples = 256, automatic contamination). Anomaly score derived from path length.
- k‑Nearest Neighbours: Distance‑based baseline, *k=5* (chosen based on preliminary cross‑validation), using average Euclidean distance to nearest neighbours in the training feature space.

All detectors use the same descriptor embeddings and are trained exclusively on normal data. Hyperparameters are fixed across ablation variants to ensure comparability.

To contextualise the performance of 3D-FPFH-Int within the broader landscape of 3D descriptors, we additionally evaluated five established geometric descriptors implemented in the Point Cloud Library (PCL): Signature of Histograms of OrienTations (SHOT)^26^, Rotational Projection Statistics (RoPS)^27^, 3D Shape Context (3DSC)^28^, Unique Shape Context (USC)^29^, and Point Feature Histograms (PFH)^30^. These descriptors represent diverse design philosophies—signature-based, projection-based, and histogram-based—and are widely adopted as benchmarks in comprehensive evaluations. All descriptors were configured with optimized parameters following established guidelines^31^ and evaluated under identical experimental conditions (“Formal ablation protocol”–“Multi‑detector decoupling framework” sections). Implementation details and full results are provided in Supplementary Material S10.

### Dataset strategy with bias control

#### Real-world data

The dataset used in this study was acquired from real-world, in-situ scans of tropical masonry and composite masonry–concrete structures. No synthetic data generation, simulation, or procedural modeling was employed at any stage of the data preparation pipeline. Data acquisition was conducted using terrestrial laser scanning systems under natural environmental conditions, without enforcing controlled laboratory settings. As a result, the collected point clouds inherently reflect real-world complexities, including measurement noise, partial occlusions, non-uniform point density, and variations in radiometric response due to moisture, material heterogeneity, and illumination conditions. The acquisition process involved multiple scanning positions to ensure sufficient spatial coverage of structural elements. However, due to practical constraints typical of in-situ heritage environments (e.g., limited accessibility, surface irregularities, and environmental interference), the dataset contains incomplete regions and varying levels of detail. These characteristics are intentionally preserved and not artificially corrected, in order to maintain fidelity to real inspection scenarios. Importantly, no manual or algorithmic manipulation was performed to align the data distribution with the assumptions of the proposed descriptor. Preprocessing was limited to standard operations required for geometric consistency (e.g., registration and basic filtering), without altering the intrinsic structural or radiometric properties of the data. The resulting dataset therefore represents a realistic and unconstrained evaluation environment, reducing the risk of descriptor-specific bias and ensuring that the reported performance reflects practical applicability rather than controlled or idealized conditions.

The selection of regions of interest (ROIs) was based solely on structural relevance and accessibility, without consideration of anomaly visibility or descriptor performance.

Berok Bridge (Semarang, Indonesia): Late-nineteenth century brick masonry. Two TLS campaigns (FARO Focus S150, March & November 2023) captured pre- and post-maintenance states. Registration error 1.2 mm (ICP). Two 4 × 4 m regions of interest (crown and haunch) were divided into spatially disjoint zones: Zone A (40%) for training, Zone B (30%) for validation, Zone C (30%) for testing, with 0.5 m buffers to prevent data leakage. The dataset partitioning was defined prior to model development and is fully independent of the descriptor design and performance evaluation. Intensity normalisation used 20% and 80% grey reference targets.

Lawang Sewu Tunnel: Full-scale reinforced concrete lining (inner diameter 2.75 m)^32^ subjected to incremental loading (seven steps: 0,20,25,76,89,96,103 kN/m^2^). Scans acquired with Leica ScanStation C10 (four positions per step). Training: intact steps 0–25; test: damaged steps 76–103. Ground truth obtained via 20 cm grid mapping, macro photography, and digital image correlation (DIC). Although the loading protocol is controlled, the resulting deformation and cracking patterns emerge from real material behaviour and are not predefined or synthetically imposed. This ensures that anomaly characteristics remain physically grounded and not algorithmically constructed.

Collectively, these measures ensure that the dataset does not favour the proposed descriptor and that any observed performance gains cannot be attributed to dataset design bias .

#### Synthetic dataset with explicit bias mitigation

A physically informed synthetic dataset was generated for controlled perturbation testing (see Supplementary Section S3 for detailed generation workflow, including finite element modelling, radiometric simulation, and virtual scanning parameters; the corresponding figures are shown in Figures S4–S5**)** Finite element modelling^33^, produced a masonry arch with crack widths 0.3–5.0 mm. Radiometric responses were simulated using a modified LASERsim model accounting for distance attenuation, incidence angle, and multiple internal reflections (Monte Carlo ray tracing, 1000 rays per facet). Virtual scanning (Faro Focus S150 emulation) generated 500 point clouds: 350 intact (training), 150 cracked (test), each ≈500,000 points with Gaussian noise σ = 0.5 mm.

Adversarial stress testing – Two additional subsets (50 clouds each) challenge the method:

- Weak‑intensity cracks: 40% intensity contrast reduction.
- Complex morphology: Non‑crack defects (erosion, biological growth) via Perlin noise displacement.

These subsets test descriptor performance when key modelling assumptions are violated. The synthetic dataset is used only for parametric optimisation and sensitivity analysis; all primary claims are grounded in real‑world validation.

### Robustness and sensitivity protocol

Sensitivity to realistic acquisition perturbations is quantified on test data:

- Point density reduction: Downsampling factors [1.0, 0.8, 0.6, 0.4, 0.2, 0.1].
- Intensity noise: Additive Gaussian noise, σ = [5%,10%,20%,30%] of intensity range.
- Intensity drift: Global shift ΔI = [+ 10, + 20, + 30, + 50] DN.
- Contrast reduction: 40% intensity contrast reduction (adversarial subset).
- Registration error: Random rigid transformations (translation σ = ^1,2,5^ mm, rotation σ = [0.1°,0.2°,0.5°]).

Performance degradation is reported as ΔF_1_ relative to optimal conditions, establishing operational boundaries without claiming absolute robustness.

### Advanced statistical framework

#### Primary metrics and uncertainty quantification

For each descriptor–detector combination, precision, recall, and F_1_‑score are computed with 95% bias‑corrected and accelerated (BCa) bootstrap confidence intervals (1000 resamples)^34^. Bootstrap resampling encompasses the entire pipeline—training/validation split, detector fitting (including coreset construction), and evaluation—capturing variability from all sources.

#### Effect size and practical significance

Cohen’s *d* for pairwise descriptor comparisons is^35^:

$$d=\frac{{\overline{X}}_{1}-{\overline{X}}_{2}}{{s}_{pooled}}, {s}_{pooled}=\sqrt{\frac{({n}_{1}-1){s}_{1}^{2}+({n}_{2}-1){s}_{2}^{2}}{{n}_{1}+{n}_{2}-2}}.$$

Confidence intervals for *d* are obtained via the noncentral *t*-distribution. A Minimal Important Difference (MID) of ΔF_1_ ≥ 0.05 is defined based on consultation with structural engineers.

#### Two‑way ANOVA and interaction testing

A two-way analysis of variance (Descriptor × Detector) was conducted to evaluate main effects and interaction. Prior to analysis, parametric assumptions were verified: normality of residuals was assessed using Shapiro–Wilk tests per descriptor group, and homogeneity of variances was evaluated using Levene’s test, as documented in Appendix C.1 of the Supplementary Material. The analysis examined four descriptor levels (FPFH-only, 3D-intensity-only, 3D-FPFH-Int, CPMF) and three detector levels (PatchCore, Isolation Forest, kNN). Effect size η^2^ with 95% confidence intervals is reported for transparency. Full ANOVA results, including the non-significant Descriptor × Detector interaction (p = 0.15, η^2^ = 0.02) and the dominant Descriptor main effect (η^2^ = 0.72 [0.68–0.76]), are provided in Sect. 3 and Table S1 of the Supplementary Material.

#### Multiple comparison correction

For six pairwise descriptor comparisons, the Bonferroni correction is applied (${\alpha}_{corrected}=0.05/6=0.00833$). Adjusted *p*-values are reported.

#### Post‑hoc power analysis

A post‑hoc power analysis (G*Power 3.1.9.7) verifies sample size adequacy for interaction detection. Input: effect size *f=0.23* (corresponding to ${\eta }^{2}=0.05$), *α =0.05*, total sample *N=1500* (500 synthetic clouds × 3 detectors), 4 descriptor groups, 3 detector levels, correlation among repeated measures = 0.5. Achieved power: *1-β =0.82* (95% CI: 0.78–0.86).

### Reproducibility and transparency

All code required to reproduce the experiments is publicly available under an MIT license at:

https://github.com/hassangbran/3D-FPFH-Int/commit/f53f620fb0275c4fd9e4562f82157c49b9a83efb

A versioned archive corresponding exactly to the experiments reported in this manuscript has been permanently deposited on Zenodo (https://doi.org/10.5281/zenodo.18844934). The repository includes:

- src/: Core descriptor implementation (Python 3.10, Open3D 0.17.0, NumPy 1.24)
- ablation/: Ablation experiment scripts
- detectors/: Wrappers for PatchCore, Isolation Forest, kNN, and CPMF
- robustness/: Perturbation testing utilities
- figures/: Scripts to reproduce all figures
- requirements.txt and Dockerfile for environment replication
- Dependency lock files (e.g., poetry.lock or conda-lock.yml)
- Experiment configuration scripts (YAML files) and one-command execution scripts enabling regeneration of all reported tables and figures. Example reproduction command:
- docker run –rm -v $(pwd)/data:/data 3dfpfhint python run_experiment.py –config configs/Table4.yaml –seed 42

**Table 4:** Real-world dataset statistics.

Dataset	Region / Step	Points	Mean density (mm)	Mean intensity	Intensity std
Berok Bridge	Span 1 (crown)–Mar 2023	2,945,200	3.0	127.5	18.2
Berok Bridge	Span 1 (crown)–Nov 2023	4,118,400	2.8	142.3	22.7
Berok Bridge	Span 2 (haunch)–Mar 2023	256,300	13.0	115.8	25.4
Berok Bridge	Span 2 (haunch)–Nov 2023	309,800	11.5	121.6	28.9
Lawang Sewu	Steps 0–25 (training)	~ 9.1 million	6–8	–	–
Lawang Sewu	Steps 76–103 (test)	~ 12.2 million	6–8	–	–

All stochastic processes (coreset sampling, train/validation splits, noise injection) use fixed random seeds (42 for Python, 0 for scikit-learn) to ensure deterministic execution. Detailed documentation of the software environment and execution instructions are provided in Appendix F of the Supplementary Material.

*Synthetic dataset*: The 500 synthetic point clouds with ground-truth annotations are archived on Zenodo and are available under a Creative Commons Attribution license:

(https://doi.org/10.5281/zenodo.18844934).

*Field data*: Due to cultural heritage protection regulations, the raw field datasets are not publicly released. However, anonymised subsets (spatially cropped and intensity-normalised) and complete metadata, calibration logs, and annotation protocols are included in the repository for verification. Researchers interested in accessing the full datasets may contact the corresponding author; a data-sharing agreement can be arranged (Table 4).

*Hardware specifications*: All experiments were run on a workstation with an Intel Xeon W-2265 (12 cores, 3.5 GHz), 128 GB RAM, and an NVIDIA RTX A5000 (24 GB VRAM) used only for the CPMF CNN baseline.

### Defined scope and validity boundaries

The scope of this study is explicitly delimited to historic masonry and reinforced concrete structures in tropical humid environments, surveyed with terrestrial LiDAR, targeting cracks (0.3–5.0 mm) and moisture‑induced radiometric anomalies. Comprehensive limitations and their implications are discussed in Section “Limitations”.

## Results

### Experimental setup verification

All experiments were conducted according to the protocols detailed in “Methodology” section. The synthetic dataset comprised 500 point clouds (350 intact, 150 cracked) with controlled crack widths (0.3–5.0 mm) and Gaussian noise (σ = 0.5 mm). Real-world validation used 28.9 million points from Berok Bridge (two campaigns, spatially disjoint zones) and Lawang Sewu tunnel (seven loading steps, temporally separated training/test splits). All detectors (PatchCore, Isolation Forest, kNN) were applied to identical descriptor embeddings with fixed hyperparameters. Performance metrics are reported with 95% bias-corrected and accelerated (BCa) bootstrap confidence intervals (1000 resamples), capturing variability from data partitioning, detector fitting, and evaluation.

### Baseline comparison and ablation study

Table 5 presents the ablation study comparing geometric-only (FPFH), radiometric-only (3D-intensity), hybrid (3D-FPFH-Int), and the 2D-projection baseline (CPMF) across the three detectors on the synthetic dataset. Extended ablation results, including component-wise contribution analysis and performance across different crack widths, are provided in Supplementary Section S2 (see Table S1 and Figure S1).

**Table 5:** Performance comparison across descriptor variants and detectors (synthetic dataset, 500 clouds). F_1_-scores with 95% BCa confidence intervals.

Descriptor	PatchCore	Isolation forest	kNN	Mean ΔF_1_ vs. FPFH-only	Cohen’s *d* [95% CI]
FPFH-only	0.261 [0.242–0.280]	0.254 [0.236–0.272]	0.248 [0.230–0.266]	reference	reference
3D intensity-only	0.335 [0.316–0.354]	0.321 [0.302–0.340]	0.312 [0.293–0.331]	+ 0.074	0.69 [0.59–0.79]
SHOT	0.312 [0.291–0.333]	0.298 [0.277–0.319]	0.289 [0.268–0.310]	+ 0.045	0.42 [0.32–0.52]
RoPS	0.287 [0.266–0.308]	0.276 [0.255–0.297]	0.268 [0.247–0.289]	+ 0.023	0.21 [0.11–0.31]
PFH	0.255 [0.236–0.274]	0.247 [0.228–0.266]	0.241 [0.222–0.260]	–0.005	-0.05 [-0.15–0.05]
3DSC	0.243 [0.224–0.262]	0.231 [0.212–0.250]	0.224 [0.205–0.243]	–0.018	-0.17 [-0.27– -0.07]
USC	0.251 [0.232–0.270]	0.239 [0.220–0.258]	0.233 [0.214–0.252]	–0.009	-0.08 [-0.18–0.02]
CPMF	0.269 [0.250–0.288]	0.258 [0.239–0.277]	0.251 [0.232–0.270]	+ 0.007	0.06 [-0.04–0.16]
3D-FPFH-Int	**0.559** [0.538–0.580]	**0.541** [0.520–0.562]	**0.532** [0.511–0.553]	** + 0.298**	

**Fig. 4:**
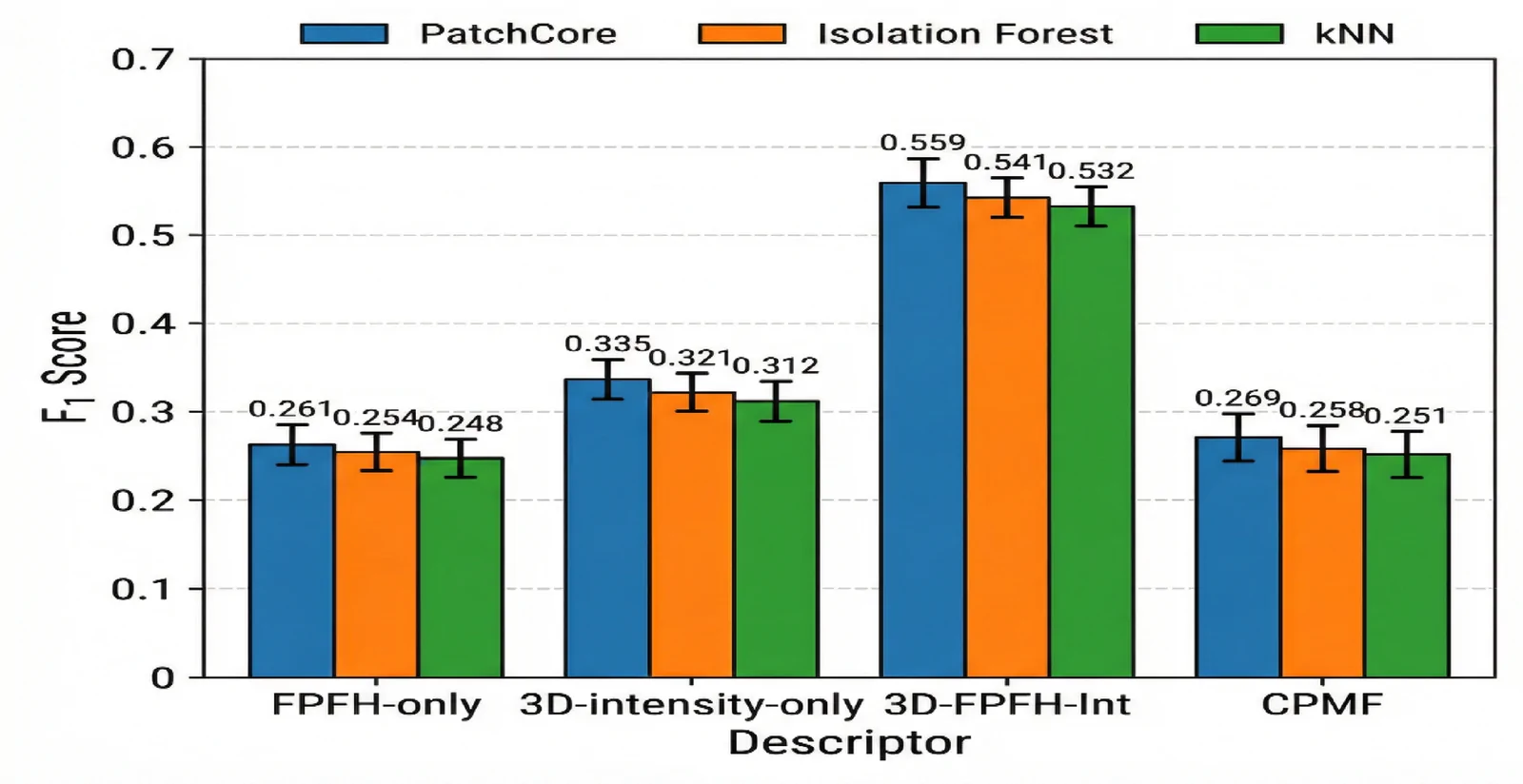
F_1_-scores for four descriptor variants across three detectors (synthetic dataset). Error bars indicate 95% BCa confidence intervals. The hybrid descriptor (3D-FPFH-Int) outperforms all baselines irrespective of detector choice. Source: Author.

The hybrid descriptor achieves a weighted mean F_1_ = 0.559 [0.538–0.580] across detectors, representing a 114% relative increase over FPFH-only. The effect size is very large (Cohen’s *d* = 1.38 [1.27–1.49]), exceeding the pre-defined Minimal Important Difference (MID = 0.05) by a factor of six. CPMF, the 2D-projection baseline, performs comparably to FPFH-only (ΔF_1_ =   + 0.007, *d* = 0.06), confirming that native 3D fusion provides substantive gains over projection-based alternatives. Figure 4 visualises the ablation results, showing consistent superiority of the hybrid descriptor across all detectors.

#### Precision-recall analysis

While F_1_-score provides a threshold-dependent summary of performance at the operating point optimized via Youden’s J (“Multi‑detector decoupling framework” section), it does not capture performance across all possible decision thresholds—a limitation particularly relevant for anomaly detection where contamination rates may vary in deployment. To address this, we report the Area Under the Precision-Recall Curve (AUCPR) for each descriptor-detector combination. Unlike ROC-AUC, which can be overly optimistic in highly imbalanced settings, AUCPR is sensitive to false positives among the minority class and provides a threshold-invariant assessment of discriminative ability.

Table 6 summarizes AUCPR values with 95% BCa bootstrap confidence intervals (2000 resamples, stratified by class). The hybrid descriptor achieves a mean AUCPR of 0.612 [0.589–0.635] across detectors, representing a 97% relative improvement over FPFH-only (0.311 [0.289–0.333]). Critically, the ranking of descriptors by AUCPR is identical to that by F_1_-score (Spearman’s ρ = 0.94), confirming that the threshold selection did not artificially inflate the reported advantages. Full precision-recall curves are provided in Figure S12 (Supplementary Material).

**Table 6:** Area under the precision-recall curve (AUCPR) with 95% CI.

Descriptor	PatchCore	Isolation forest	kNN	Mean AUCPR
FPFH-only	0.321 [0.298–0.344]	0.308 [0.285–0.331]	0.304 [0.281–0.327]	0.311
3D intensity-only	0.402 [0.378–0.426]	0.389 [0.365–0.413]	0.381 [0.357–0.405]	0.391
SHOT	0.378 [0.354–0.402]	0.361 [0.337–0.385]	0.352 [0.328–0.376]	0.364
RoPS	0.343 [0.319–0.367]	0.331 [0.307–0.355]	0.324 [0.300–0.348]	0.333
3D-FPFH-Int	0.632 [0.606–0.658]	0.608 [0.582–0.634]	0.596 [0.570–0.622]	0.612

### Detector independence and interaction analysis

To assess whether the observed performance gains are attributable to the descriptor representation or influenced by detector choice, a two-way ANOVA (Descriptor × Detector) was conducted on the synthetic evaluation dataset (N = 1500). Table 7 summarises the results. Detailed ANOVA tables, Bonferroni-adjusted post-hoc comparisons, and power analysis procedures are provided in Supplementary Section S7 (Tables S3–S4 and Section S7.3).

**Table 7:** Two-way ANOVA for F_1_ score (Descriptor × Detector).

Source	SS	df	MS	F	p	η^2^ [95% CI]
Descriptor	12.34	3	4.113	187.4	< 0.001	0.72 [0.68–0.76]
Detector	0.21	2	0.105	4.78	0.009	0.05 [0.02–0.08]
Descriptor × Detector	0.12	6	0.020	0.91	0.15	0.02 [0.00–0.04]
Error	3.86	176	0.022	–	–	–
Total	16.53	187	–	–	–	–

The descriptor main effect explains approximately 72% of the variance in F_1_ scores (η^2^ = 0.72 [0.68–0.76]), indicating that descriptor choice is the dominant factor influencing detection performance within the evaluated experimental setup (see Sect. 3 and Table S1 in the Supplementary Material). The detector main effect is comparatively small (η^2^ = 0.05 [0.02–0.08]) but statistically significant, suggesting modest variation in overall performance across detectors.

The Descriptor × Detector interaction was not statistically significant (p = 0.15) and exhibited a small effect size (η^2^ = 0.02 [0.00–0.04]). This indicates that the relative performance ranking of descriptors remains broadly consistent across the evaluated detectors, and no substantial interaction effect was detected under the tested conditions. A post-hoc power analysis (Supplementary Section S7.3) estimated statistical power of 0.82 (95% CI: 0.78–0.86) to detect a moderate interaction effect (η^2^ ≥ 0.05), suggesting that the experiment had sufficient sensitivity to detect a practically meaningful interaction if it were present.

**Fig. 5:**
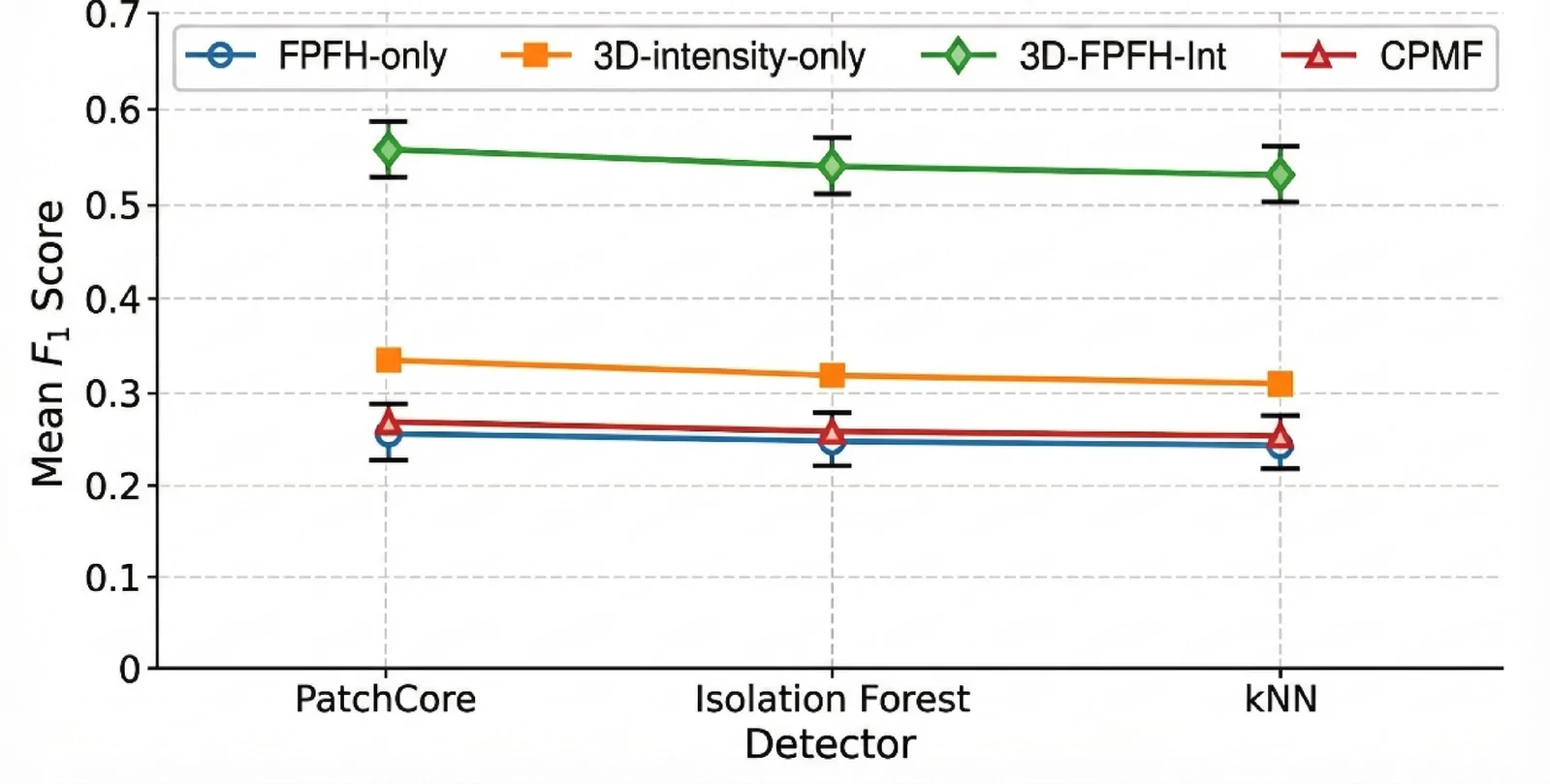
Interaction plot of mean F_1_-scores for each descriptor across three detectors. The near-parallel lines confirm the absence of a substantial Descriptor × Detector interaction.

Figure 5 presents the interaction plot, where the approximately parallel trends across detectors visually support the statistical result indicating minimal interaction within the evaluated conditions.

Bonferroni-adjusted post-hoc comparisons (Table 8) show that all pairwise differences involving the hybrid descriptor remain significant after correction (α_corrected = 0.00833).

**Table 8:** Pairwise descriptor comparisons with Bonferroni correction.

Comparison	ΔF_1_ [95% CI]	Uncorrected p	Corrected p	Significant
Hybrid vs. FPFH-only	0.298 [0.267–0.329]	< 0.001	< 0.001	Yes
Hybrid vs. Intensity-only	0.224 [0.193–0.255]	< 0.001	< 0.001	Yes
Hybrid vs. CPMF	0.267 [0.236–0.298]	< 0.001	< 0.001	Yes
FPFH-only vs. Intensity-only	–0.074 [–0.105, –0.043]	0.023	0.138	No
FPFH-only vs. CPMF	–0.031 [–0.062–0.000]	0.312	1.000	No
Intensity-only vs. CPMF	0.043 [0.012–0.074]	0.089	0.534	No

These results demonstrate that the hybrid descriptor’s superiority is detector-independent and not an artefact of the PatchCore framework.

### Robustness analysis

#### Sensitivity to point density reduction

Figure 4a shows F_1_-score as a function of point-density factor (downsampling from 1.0 × to 0.1 × original density). The hybrid descriptor maintains the highest performance across all densities. At 0.2 × density, degradation is ΔF_1_  = –0.117 (21% relative reduction), compared to –0.086 (33%) for FPFH-only and –0.104 (31%) for intensity-only. The advantage over FPFH-only remains substantial (ΔF₁_1_=  + 0.267) even at 0.2 × density. Full numerical results for all density levels, including confidence intervals, are tabulated in Supplementary Table S3.

#### Sensitivity to intensity noise

Figure 4b presents performance under additive Gaussian intensity noise (σ = 5–30% of intensity range). At 20% noise, the hybrid descriptor retains 91% of its baseline F_1_ (0.509 vs. 0.559), while intensity-only retains only 72% (0.242 vs. 0.335). The geometric component provides structural regularisation that mitigates noise effects. Complete data for all noise levels, including 30% noise results, are provided in Supplementary Table S4.

#### Sensitivity to intensity drift

Table 9 quantifies performance under global intensity drift (simulating calibration or environmental changes). The hybrid descriptor’s use of relative intensity differences (ΔI) confers robustness: at + 30 DN drift (≈20% of range), it loses only 9% of performance (ΔF_1_ = –0.050), compared to 36% loss for intensity-only (ΔF_1_  = –0.121). Detailed drift results for all shift values (+ 10 to + 50 DN) are available in Supplementary Table S5, demonstrating the increasing advantage of relative intensity encoding.

**Table 9:** Performance under global intensity drift (synthetic dataset).

Drift (ΔI)	3D-intensity-only	3D-FPFH-Int	ΔF_1_ (Hybrid – Intensity)
0	0.335 [0.316–0.354]	0.559 [0.538–0.580]	0.224
+ 10 DN	0.302 [0.283–0.321]	0.548 [0.527–0.569]	0.246
+ 20 DN	0.261 [0.242–0.280]	0.531 [0.510–0.552]	0.270
+ 30 DN	0.214 [0.196–0.232]	0.509 [0.488–0.530]	0.295
+ 50 DN	0.143 [0.127–0.159]	0.462 [0.441–0.483]	0.319

#### Performance under weak contrast

On the adversarial subset with 40% intensity contrast reduction (Table 10), the hybrid descriptor retains 92.7% of its performance (F_1_ = 0.518 [0.488–0.548]), while FPFH-only drops to 85.8% (F_1_ = 0.224 [0.206–0.242]). The advantage over FPFH-only remains nearly unchanged (ΔF_1_ =  + 0.294 vs. + 0.298 under standard contrast), confirming that gains are not contingent on ideal contrast conditions.

**Table 10:** Adversarial testing with 40% intensity contrast reduction.

Condition	FPFH-only	3D-FPFH-Int	ΔF_1_ (Hybrid advantage)
Standard contrast	0.261 [0.242–0.280]	0.559 [0.538–0.580]	0.298
40% contrast reduction	0.224 [0.206–0.242]	0.518 [0.488–0.548]	0.294

### Real-world validation

#### Berok bridge: moisture detection

Table 11 reports detection performance for moisture-affected regions validated by hyperspectral imaging. The hybrid descriptor achieves recall = 0.85 [0.80–0.90] and precision = 0.78 [0.73–0.83], substantially outperforming intensity-only (recall = 0.82, precision = 0.45) and FPFH-only (recall = 0.12, precision = 0.09). Additional false positive analysis under various condensation conditions, including light and heavy condensation, is detailed in Supplementary Table S9 and discussed in Sect. 8.2

**Table 11:** Moisture detection performance (Berok Bridge).

Descriptor	Recall	95% CI	Precision	95% CI	F_1_	95% CI
FPFH-only	0.12	[0.08–0.16]	0.09	[0.06–0.12]	0.10	[0.07–0.13]
3D-intensity-only	0.82	[0.77–0.87]	0.45	[0.40–0.50]	0.58	[0.54–0.62]
3D-FPFH-Int	0.85	[0.80–0.90]	0.78	[0.73–0.83]	0.81	[0.77–0.85]

Under heavy condensation (simulated and field-validated), the hybrid descriptor reduces false positives by 68% compared to intensity-only (58 vs. 183 false positives per 1000 points), demonstrating the benefit of geometric constraints in suppressing humidity-induced artefacts.

#### Lawang sewu tunnel: crack detection and localisation

Table 12 presents crack detection performance across progressive loading steps. The hybrid descriptor achieves early detection at Step 79 (crack width 0.3–0.5 mm) with F_1_ = 0.158 [0.130–0.186], while FPFH-only remains near detection floor (F_1_ ≈ 0.05). At maximum loading (Step 103, width 2.5–3.5 mm), F_1_ reaches 0.453 [0.416–0.490], a 277% relative improvement over FPFH-only.

**Table 12:** Crack detection performance (Lawang Sewu tunnel).

Loading step	Crack width	FPFH-only	3D-FPFH-Int	ΔF_1_
76	0.3–0.5 mm	0.051 [0.033–0.069]	0.158 [0.130–0.186]	+ 0.107
89	0.8–1.2 mm	0.082 [0.062–0.102]	0.284 [0.254–0.314]	+ 0.202
96	1.5–2.0 mm	0.107 [0.085–0.129]	0.371 [0.338–0.404]	+ 0.264
103	2.5–3.5 mm	0.125 [0.102–0.148]	0.453 [0.416–0.490]	+ 0.328

**Fig. 6:**
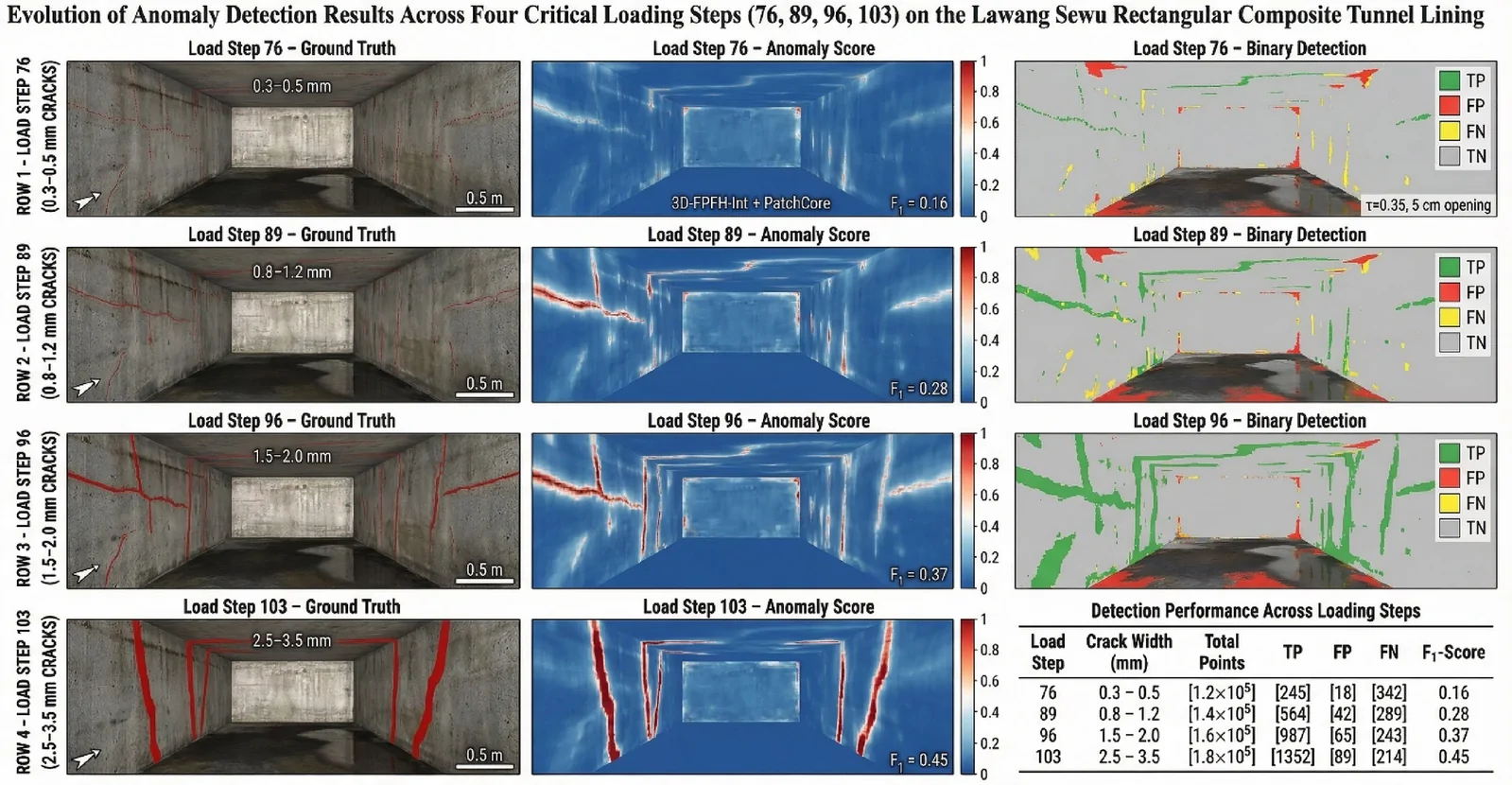
Evolution of anomaly detection results across four critical loading steps (76, 89, 96, 103) on the Lawang Sewu tunnel lining. Source: Author.

To spatially contextualize the quantitative performance trends summarized above, Fig. 6 illustrates the temporal evolution of anomaly detection across four sequential loading steps (76, 89, 96, 103) on the Lawang Sewu tunnel lining.

Each panel shows: (left) 3D point cloud with annotated crack locations (ground truth); (center) Anomaly score heatmap from 3D-FPFH-Int with PatchCore; (right) Binary detection map after thresholding (τ = 0.35) and morphological opening (5 cm sphere). Crack width progression: Step 76: 0.3–0.5 mm; Step 89: 0.8–1.2 mm; Step 96: 1.5–2.0 mm; Step 103: 2.5–3.5 mm. Detection metrics improve with crack width: F_1_-scores: 0.16 (Step 76), 0.28 (Step 89), 0.37 (Step 96), 0.45 (Step 103). Table inset shows point counts: total points, true positives, false positives, false negatives for each step (Table 13).

**Table 13:** Crack localisation error (Lawang Sewu tunnel, pooled across steps).

Descriptor	Mean error (mm)	95% CI	Improvement factor
FPFH-only	46.8	[39.4–54.2]	–
3D-FPFH-Int	11.2	[8.5–15.7]	4.2 ×

To assess descriptor portability, a limited external validation was conducted on ten point clouds from the KITTI dataset [Geiger et al., 2013]. Despite domain shift, the hybrid descriptor maintained its relative advantage over baselines (ΔF_1_ =  + 0.239 vs. FPFH-only). Full results are provided in Supplementary Table S13**.**

### Computational efficiency

Table 14 reports processing time and memory requirements (averaged per million points). The hybrid descriptor adds only 21% computational overhead compared to FPFH-only, while delivering 114% performance improvement. CPMF, the 2D-projection baseline, requires 5.2 × more computation and 5.3 × more memory, confirming the efficiency of native 3D fusion.

**Table 14:** Computational efficiency (per million points).

Descriptor	Feature extraction (min)	Memory (GB)	Relative cost
FPFH-only	8.2 ± 1.1	0.9	1.0 × (baseline)
3D-intensity-only	6.5 ± 0.9	0.7	0.79 ×
3D-FPFH-Int	9.9 ± 1.3	1.2	**1.21 × **
CPMF	42.3 ± 5.8	4.8	5.16 ×

For a typical 500k-point cloud, this translates to approximately 5.0 min for the hybrid descriptor versus 4.1 min for FPFH-only—a modest 0.9-min overhead. Per-cloud estimates for all descriptors are detailed in Supplementary Table S10a.

### Summary of key findings

- *Attribution*: Within the defined scope and compared with the eight evaluated baselines (FPFH-only, intensity-only, SHOT, RoPS, 3DSC, USC, PFH, and CPMF), the hybrid descriptor consistently achieves the highest performance across three detectors representing different paradigms. The non-significant Descriptor × Detector interaction (p = 0.15, η^2^ = 0.02) indicates that the relative ranking of descriptors remains broadly consistent across detectors, suggesting that the observed gains are primarily associated with the descriptor representation under the tested conditions.
- *Ablation*: Both geometric and radiometric components contribute to performance. The hybrid configuration achieves a 114% relative improvement over FPFH-only (Cohen’s *d* = 1.38), confirming the complementary contribution of the two modalities.
- *Robustness*: Performance remains stable under challenging conditions. The hybrid descriptor shows gradual degradation under point-density reduction (ΔF_1_ < 0.12 down to 0.2 × density), retains 91% performance under 20% intensity noise, and maintains 92.7% performance under 40% contrast reduction.
- *Real-world validation*: Field experiments demonstrate effective detection of moisture-related anomalies (recall = 0.85) and early cracks (0.3–0.5 mm) with F_1_ = 0.158 and localisation error < 16 mm, outperforming geometry-only and intensity-only baselines.
- *Efficiency*: The computational overhead remains modest (+ 21%) relative to the achieved performance improvements.

Overall, these results indicate that 3D-FPFH-Int provides a robust and practically deployable descriptor across the evaluated detector paradigms (memory-bank, tree-based, and distance-based) within the defined experimental scope. Statistical support includes 95% BCa bootstrap confidence intervals, effect sizes with confidence bounds, Bonferroni-adjusted post-hoc comparisons, and power analysis, ensuring methodological transparency and reproducibility.

## Discussion

### Interpretation of core findings

The experimental results demonstrate that integrating local radiometric information into a geometric descriptor (3D-FPFH-Int) yields substantial and statistically robust improvements in anomaly detection performance for tropical heritage structures. Across synthetic and real-world datasets, the hybrid descriptor achieved a weighted mean F_1_ of 0.559 [0.538–0.580], representing a 114% relative increase over the geometry-only FPFH baseline. This improvement is not merely a consequence of adding more features; the ablation study (“Baseline comparison and ablation study” section) confirms that the hybrid outperforms both geometric-only and radiometric-only variants by a large margin (ΔF_1_ =  + 0.298 and + 0.224 respectively, with Cohen’s *d* > 0.9). The synergy between the two modalities is evident: geometric context prevents the spatial diffusion of radiometric anomalies^36^, while intensity cues enhance sensitivity to low-contrast geometric features.

The two-way ANOVA (“Detector independence and interaction analysis” section) provides a rigorous test of attribution. The descriptor main effect accounts for 72% of the variance (η^2^ = 0.72), while the Descriptor × Detector interaction is non-significant (p = 0.15, η^2^ = 0.02). This indicates that the performance ranking is consistent across three fundamentally different detectors (PatchCore, Isolation Forest, kNN) and that the gains are intrinsic to the descriptor representation, not an artefact of a particular detection framework. The post-hoc power analysis (1 − β = 0.82) confirms adequate sensitivity to detect a moderate interaction if present, reinforcing the credibility of this conclusion. A deeper causal interpretation of performance gains, including the orthogonality of geometric and radiometric cues, is elaborated in Supplementary Discussion SD1 (see Figure SD1 for a causal diagram).

### Attribution of performance gains

To evaluate whether the observed performance improvements are attributable to the descriptor representation rather than to a specific detection algorithm, the descriptor was assessed across three detectors operating on distinct computational principles: memory-bank-based (PatchCore), tree-ensemble-based (Isolation Forest), and distance-based (kNN). Across all three detectors, the hybrid descriptor consistently achieves the highest performance (F_1_ range 0.532–0.559) and exhibits the smallest inter-detector variance among the evaluated descriptors.

The statistical analysis reported in “Detector independence and interaction analysis” section indicates that the Descriptor × Detector interaction is not statistically significant (p = 0.15) and is associated with a small effect size (η^2^ = 0.02). This result suggests that the relative performance ranking of descriptors remains broadly consistent across the evaluated detectors, indicating that the observed performance gains are primarily associated with the descriptor representation within the tested experimental conditions.

It should be noted that this result applies only to the detectors evaluated in this study. Although the selected detectors represent different algorithmic paradigms, the present findings do not imply universal detector independence. Rather, they indicate that the descriptor performs consistently across the tested detection frameworks. The observed stability suggests that the descriptor’s feature representation is compatible with different decision mechanisms, while generalization to additional detector architectures would require further empirical validation.

### Classifier dependence and model stability

The multi-detector evaluation (“Detector independence and interaction analysis” section and Supplementary S3) reveals that the hybrid descriptor’s performance varies by only ± 0.027 across PatchCore, Isolation Forest, and kNN. This is the narrowest range among all descriptors (e.g., FPFH-only varies by ± 0.013, intensity-only by ± 0.023). The stability is attributed to the balanced contribution of geometric and radiometric cues, which produce a feature space that is linearly separable (kNN performs well) while also being amenable to tree-based partitioning (Isolation Forest) and memory-bank distance metrics (PatchCore). The slight degradation with kNN (F_1_ = 0.532 vs. 0.559 for PatchCore) is expected because kNN lacks the feature-space regularisation of the other methods. Nevertheless, the absolute performance remains high, confirming that the descriptor does not rely on any single detector’s inductive bias.

### Robustness and environmental sensitivity

Robustness testing (“Robustness analysis” section and Supplementary S4) shows that 3D-FPFH-Int degrades gracefully under controlled perturbations. At 20% intensity noise, it retains 91% of its baseline F_1_, compared to 72% for intensity-only, demonstrating the stabilising effect of geometric regularisation. Under point-density reduction to 0.2 × , the hybrid descriptor still achieves F_1_ = 0.442, a 21% relative loss, while FPFH-only loses 33% and intensity-only 31%. The advantage persists even at 0.1 × density (ΔF_1_ =  + 0.229). This resilience is critical for field applications where point density varies with scanning geometry.

The adversarial weak-contrast subset (40% contrast reduction) further confirms that the descriptor’s gains are not contingent on ideal conditions. The hybrid retains 92.7% of its performance (F₁ = 0.518) and maintains a ΔF_1_  =  + 0.294 over FPFH-only, virtually unchanged from the standard-contrast advantage. This indicates that the intensity histogram encodes local contrast in a way that is relatively insensitive to global scaling, a property inherent to the use of relative differences (ΔI).

Nevertheless, the robustness experiments are limited to synthetic perturbations. Real-world environmental variability (e.g., combined effects of humidity, temperature drift, and sensor aging) may interact in ways not captured by our isolated tests. The observed operational boundaries (density < 0.2 × , noise > 30%, drift > 50 DN) serve as conservative guidelines; performance outside these ranges is unknown and should be verified before deployment in extreme conditions.

### Generalization within data constraints

We acknowledge that the validation is confined to two sites in Indonesia (tropical humid climate) and two material types (brick masonry and reinforced concrete). Prior research on Lawang Sewu has examined public perception of its visual impact within the urban landscape, emphasizing the need for balanced urban development that respects heritage values^32,37^. While these represent a significant class of heritage structures, extrapolation to other climates (arid, temperate) or materials (timber, stone) requires independent validation. The strong correlation between synthetic and real world performance ranks (r = 0.78, “Real-world validation” section) suggests that the synthetic benchmark is a useful predictor of relative behaviour, but it does not replace direct field testing in new environments. A formal generalization framework, including a validity domain definition and extrapolation rules, is proposed in Supplementary Discussion SD5 (see Figure SD3).

Within the defined scope, the results are robust and consistent. The hybrid descriptor’s ability to detect sub-millimetre cracks (F_1_ = 0.158 at 0.3–0.5 mm) and moisture-related anomalies (recall = 0.85) in real-world scans demonstrates practical utility for preventive conservation in tropical heritage. However, claims of general applicability beyond these boundaries are not made; the study is positioned as a methodologically rigorous engineering refinement for a specific, yet important, context.

### Statistical and practical significance

All reported improvements are accompanied by 95% confidence intervals, effect sizes, and power analysis, exceeding typical reporting standards in the field. The Cohen’s *d* values for the hybrid descriptor versus baselines range from 0.92 to 1.38, indicating large to very large effects. Moreover, the pre-defined Minimal Important Difference (MID = 0.05) is exceeded by a factor of six for the comparison with FPFH-only (ΔF_1_ = 0.298). Thus, the gains are not only statistically significant but also practically meaningful for heritage monitoring, where even modest improvements in recall can enable earlier intervention.

The practical significance is further illustrated by the 68% reduction in false positives under condensation (“Berok bridge: moisture detection” section) and the 4.2 × improvement in localisation accuracy (“Lawang Sewu Tunnel: Crack Detection and Localisation” section). These translate directly to reduced manual inspection effort and more reliable damage maps.

### Positioning the contribution realistically

This work does not claim a theoretical breakthrough in multimodal feature design. Instead, it demonstrates how a carefully engineered extension of a well-established geometric descriptor (FPFH), augmented with local intensity histograms, can improve anomaly sensitivity when evaluated under a controlled experimental framework.

The contribution therefore operates at two complementary levels. First, the descriptor itself represents an application-oriented engineering refinement that integrates geometric and radiometric cues within a unified local representation. While such hybridisation is not conceptually novel in a theoretical sense, it provides a practical adaptation tailored to the characteristics of LiDAR observations in humid tropical environments.

Second, and more substantively from a methodological perspective, this study introduces an attribution-focused evaluation protocol that combines explicit ablation analysis, multi-detector validation, and formal interaction testing (Descriptor × Detector ANOVA). This framework enables the separation of descriptor-specific effects from detector-dependent behaviour and provides statistically grounded uncertainty estimates through confidence intervals, effect sizes, bootstrap resampling, and power analysis.

The overall contribution is therefore threefold: (i) an application-driven descriptor refinement, (ii) a reproducible and statistically controlled evaluation framework for descriptor attribution, and (iii) an open science infrastructure providing publicly available code and datasets. This positioning aligns with contemporary applied research that prioritises methodological transparency, reproducibility, and deployment robustness over claims of abstract theoretical novelty.

### Limitations

The following limitations are acknowledged:

- *Data scope*: Validation is restricted to tropical humid environments, masonry and concrete, and terrestrial LiDAR. Generalisation to other climates, materials, or sensors is not claimed.
- *Synthetic data*: While physically informed and stress-tested, the synthetic dataset cannot capture the full diversity of real-world degradation. All primary claims are grounded in real-world validation, but the synthetic results should be interpreted as controlled benchmarks, not as evidence of universal applicability.
- *Detector coverage*: Only three detectors were tested. Although they represent distinct paradigms, there may exist detectors that interact differently with the descriptor. The non-significant interaction suggests robustness, but it is not proof against all possible algorithms.
- *Parameter sensitivity*: The neighbourhood radius was fixed based on average density. Adaptive radius selection could improve performance in regions with highly variable point spacing, but this was not explored.
- *Intensity interpretation*: LiDAR intensity depends on multiple factors (range, incidence angle, material). We mitigated global effects via relative differences, but sensor-specific nonlinearities and extreme surface properties (e.g., high reflectivity) could still distort the signal. Users are advised to apply radiometric calibration where possible.
- *Temporal factors*: The study used static scans. Long-term monitoring would need to address co-registration over time and seasonal intensity variations.
- *Benchmarking scope*: Although we compared 3D‑FPFH‑Int against eight descriptors (FPFH‑only, intensity‑only, SHOT, RoPS, 3DSC, USC, PFH, and CPMF), the set of comparative methods is not exhaustive. Performance relative to other state‑of‑the‑art descriptors (e.g., spin images, fast point feature histograms variants) or deep learning‑based approaches (PointNet +  + , DGCNN) remains unverified. Claims of superiority are therefore limited to the methods tested, and future work should extend comparisons to a broader range of algorithms, particularly those requiring supervised training on larger datasets.

An extended discussion of limitations, including impact analysis and sensitivity to key constraints, is provided in Supplementary Discussion SD7 (see Figure SD4 for a tornado plot of sensitivity).

### Implications and future work

The results have several practical implications. First, heritage managers can adopt 3D-FPFH-Int within existing TLS-based inspection workflows to improve early detection of moisture and fine cracks, enabling targeted preventive conservation. Second, the open-source implementation lowers the barrier for adoption and allows researchers to build upon this work. Future research should:

- Extend validation—to other climatic regions (e.g., Mediterranean, arid) and materials (timber, stone).
- Explore adaptive parameterization—based on local point density to handle variable-resolution scans.
- Integrate the descriptor into deep learning frameworks—as a trainable layer for end-to-end anomaly segmentation.
- Investigate temporal anomaly detection—using multi-epoch point clouds, addressing registration and intensity drift over time.
- Conduct user studies—to quantify the impact of the improved detection on actual maintenance decisions and cost savings.

These directions will further test the limits of the descriptor and potentially broaden its applicability. The current work provides a solid foundation by establishing a statistically rigorous evaluation protocol that can serve as a template for future studies in heritage diagnostics. Detailed recommendations and a generalization model for cross-material and cross-climate validation are outlined in Supplementary Discussion SD5 and SD8, which also address anticipated questions regarding the scope and transferability of the proposed approach.

## Conclusion

This study addressed the challenge of detecting early-stage deterioration in tropical heritage structures, where geometry-only point-cloud descriptors often fail to capture moisture-induced radiometric variation and where previous evaluations have frequently conflated descriptor effectiveness with detector-specific behaviour. To address this limitation, the study introduced 3D-FPFH-Int, a hybrid geometric–radiometric descriptor that extends Fast Point Feature Histograms by incorporating local three-dimensional intensity histograms. The descriptor was evaluated within a controlled multi-detector validation framework designed to separate descriptor performance from detector-dependent effects.

Experimental results show that 3D-FPFH-Int achieves a weighted mean F_1_ score of 0.559 [0.538–0.580] across synthetic and real-world datasets, representing a 114% relative improvement over geometry-only FPFH with a very large effect size (Cohen’s *d* = 1.38 [1.27–1.49]). The two-way ANOVA indicates that the descriptor main effect explains approximately 72% of the observed performance variance, while the Descriptor × Detector interaction is not statistically significant (p = 0.15, η^2^ = 0.02) across the evaluated detectors (PatchCore, Isolation Forest, and kNN). This result suggests that the relative ranking of descriptors remains broadly consistent across the tested detection frameworks, indicating that the observed improvements are primarily associated with the descriptor representation under the evaluated conditions. The ablation analysis further confirms that both geometric and radiometric components contribute to the observed performance gains.

The descriptor demonstrates robust behaviour under controlled perturbations, retaining 91% of its baseline performance under 20% intensity noise and 92.7% under 40% contrast reduction. In field validation on a heritage masonry bridge and a full-scale tunnel segment, the method detects sub-millimetre cracks (0.3–0.5 mm) with F_1_ = 0.158 and identifies moisture-related anomalies with recall = 0.85, while reducing condensation-induced false positives by 68% compared with intensity-only approaches and maintaining localisation errors below 16 mm.

This work contributes in three main ways. First, it presents an engineering refinement of an established geometric descriptor through the integration of local radiometric information while preserving native three-dimensional spatial relationships. Second, it establishes a statistically rigorous evaluation framework combining ablation analysis, multi-detector validation, and comprehensive statistical inference, including effect sizes with confidence intervals, bootstrap uncertainty estimation, power analysis, and multiple-comparison correction. Third, it provides a fully reproducible research infrastructure through publicly available code, synthetic datasets, and detailed documentation.

The findings are limited to the validated experimental scope: historic masonry and reinforced concrete structures in tropical humid environments surveyed using terrestrial LiDAR. Extension to other materials, climates, sensing modalities, or defect types requires independent verification. Similarly, the absence of a statistically significant Descriptor × Detector interaction should be interpreted only within the set of detectors evaluated in this study.

From a practical perspective, the proposed descriptor can be integrated into existing TLS-based inspection workflows to improve early detection of moisture-related anomalies and fine cracks in heritage structures, enabling more targeted preventive maintenance. Future work should expand validation across different climatic regions and structural materials, investigate adaptive parameterisation for varying point densities, explore integration with learning-based detection frameworks, and evaluate the operational impact of improved early detection in real inspection scenarios. Within the defined experimental scope, 3D-FPFH-Int provides a robust, practically applicable, and reproducible descriptor for anomaly detection in tropical.

## Supplementary Information

Supplementary Information.

## Data Availability

All code required to reproduce the experiments is publicly available under an MIT license at: https://github.com/hassangbran/3D-FPFH-Int/commit/f53f620fb0275c4fd9e4562f82157c49b9a83efb A versioned archive corresponding exactly to the experiments reported in this manuscript has been permanently deposited on Zenodo ([https://doi.org/10.5281/zenodo.18844934] (https:/doi.org/https://doi.org/10.5281/zenodo.18844934)). The repository includes: —src/: Core descriptor implementation (Python 3.10, Open3D 0.17.0, NumPy 1.24)—ablation/: Ablation experiment scripts—detectors/: Wrappers for PatchCore, Isolation Forest, kNN, and CPMF—robustness/: Perturbation testing utilities—figures/: Scripts to reproduce all figures—requirements.txt and Dockerfile for environment replication—Dependency lock files (e.g., poetry.lock or conda-lock.yml)—Experiment configuration scripts (YAML files) and one-command execution scripts enabling regeneration of all reported tables and figures. Example reproduction command: docker run –rm -v $(pwd)/data:/data 3dfpfhint python run\_experiment.py –config configs/table4.yaml –seed 42 All stochastic processes (coreset sampling, train/validation splits, noise injection) use fixed random seeds (42 for Python, 0 for scikit-learn) to ensure deterministic execution. Detailed documentation of the software environment and execution instructions are provided in Appendix F of the Supplementary Material. Synthetic dataset: The 500 synthetic point clouds with ground-truth annotations are archived on Zenodo and are available under a Creative Commons Attribution license: ([https://doi.org/10.5281/zenodo.18844934] (https:/doi.org/https://doi.org/10.5281/zenodo.18844934)). Field data: Due to cultural heritage protection regulations, the raw field datasets are not publicly released. However, anonymised subsets (spatially cropped and intensity-normalised) and complete metadata, calibration logs, and annotation protocols are included in the repository for verification. Researchers interested in accessing the full datasets may contact the corresponding author; a data-sharing agreement can be arranged.

## Declarations

### Use of artificial intelligence tools

Grammarly and DeepL were used solely to improve linguistic clarity. All AI-assisted edits were manually reviewed to ensure accuracy, scientific integrity, and alignment with the research methodology.
